# Anticolitic Effect of Berberine in Rat Experimental Model: Impact of PGE2/p38 MAPK Pathways

**DOI:** 10.1155/2020/9419085

**Published:** 2020-09-29

**Authors:** Li Jia, Kuijin Xue, Junheng Liu, Ola A. Habotta, Lianhai Hu, Ahmed E. Abdel Moneim

**Affiliations:** ^1^Department of Gastroenterology, Maternal and Child Health Care Hospital of Shandong Province, Jinan 251400, China; ^2^Department of Gastroenterology, The Affiliated Hospital of Qingdao University, Qingdao City 266000, China; ^3^Department of Forensic Medicine and Toxicology, Faculty of Veterinary Medicine, Mansoura University, Mansoura 35516, Egypt; ^4^Department of Traditional Chinese Medicine, Qilu Hospital, Cheeloo College of Medicine, Shandong University, No. 107 Wenhuaxi Road, Jinan, Shandong Province 250012, China; ^5^Department of Zoology and Entomology, Faculty of Science, Helwan University, Cairo 11795, Egypt

## Abstract

Berberine (BER), a natural isoquinoline alkaloid, has been demonstrated to have appreciable anticolitis effects. Nevertheless, the protective mechanism of BER in ulcerative colitis (UC) is barely understood. The present study was aimed at exploring the therapeutic efficacy of BER on UC in experimental colitis rat model. Rats were orally administered with BER for seven days at low and high doses (25 and 50 mg/kg/day) before AcOH intracolonic instillation. BER significantly retrieved colon inflammation and mucosal damage indicated by inhibition of macroscopic score and lessened the levels of inflammatory biomarkers (IL-1*β*, IL-6, TNF-*α*, MPO, and PGE2). Notable downregulation of mRNA expression of p38 MAPK and increased protein expression of TGF-*β* were achieved by BER treatment. The anti-inflammatory potential of BER was supported by the histopathological screening of colon mucosa. In addition, BER restored colonic antioxidant capacity through elevation of GSH level and antioxidant enzymatic activities (SOD, CAT, GPx, and GR) together with reductions of both MDA and NO levels. Marked downregulation of *Nos2* mRNA expression is accompanied by increased Nrf2 and Hmox-1 expressions in colon specimens treated by BER. Furthermore, BER exhibited noticeable antiapoptotic activities through decreasing proapoptotic proteins (Bax and caspase-3) and lessening antiapoptotic Bcl-2 protein in the colon mucosa. Based on these findings, BER may improve colitis markedly which may be mediated by its striking antioxidant, anti-inflammatory, and antiapoptotic properties.

## 1. Introduction

Inflammatory bowel disease is an umbrella term which includes mainly two subtypes, ulcerative colitis (UC) and Crohn's disease, and clinically characterized by severe abdominal pain, diarrhea, fatigue, and weight loss [1]. Of which, UC is associated with chronic relapsing nonspecific inflammation and ulcer formation in the rectum and sigmoid colon with architectural epithelial damage and mucosal barrier dysfunction [2]. The prevalence of UC is steadily increasing globally plaguing thousands of people especially in the past few years and affects the quality of life to great extent [3]. Systemically, some manifestations were associated with UC such as enteropathic arthritis, primary sclerosing cholangitis, and ocular and skin damage [4].

Extensive studies had been conducted to understand the pathogenic mechanism of UC, but its definite etiology is still unclear. Exaggerated inflammatory response and overproduction of inflammatory mediators such as tumour necrosis factor (TNF)-*α* and cytokines (interleukin (IL)-6 and IL-1*β*) play a pivotal role in colon inflammation [5]. Prostaglandin E2 (PGE2) is the main PG in the gastrointestinal tract and physiologically influences on acid and mucus secretion, blood flow, intestinal motility, and mucosal health. However, high levels of PGE2 have proinflammatory effects and are involved in progress of UC pathogenesis [6]. Overgeneration of free radical species is a main sequence of inflammatory signalling in the colon with subsequent exhaustion of cellular antioxidant capability and lipid peroxidation [7].

Nuclear factor erythroid 2-related factor 2 (Nrf2) has a principle role in protecting the cells against oxidative injury through production of phase-2 detoxifying enzymes and stress-related proteins. Also, Nrf2 signalling was reported to be involved in inflammatory reactions associated with some diseases such as colitis, gastritis, rheumatoid arthritis, and atherosclerosis [8]. Earlier investigations revealed enhanced MAPK signalling pathways in inflammatory gut diseases due to the induction of iNOS expression [9]. Therefore, MAPK and Nrf2 signalling pathways are striking therapeutic strategy to alleviate the colon inflammation.

Until now, there are no specific medications approved for treatment of UC. The drug most frequently used in UC is 5-qminosalicilic acid [2]. However, the available biologic agents revolutionized the treatment of UC improving the quality of life of thousands of people suffering from this disease. Certainly, the cost of biologic agents is high; however, it can reduce both the number of hospitalizations and the need for frequent medical visits. Natural origin treatment at the moment could only be used in parallel with the western medical treatment.

Berberine (BER) is an isoquinoline alkaloid extracted from *Hydrastis canadensis*, *Berberis aquifolium*, and *Berberis vulgaris* [10]. It possesses various pharmacologic effects including antibacterial, antiprotozoal, and anticholera toxin activities. Also, it is used for treatment of diarrhea, diabetes, hyperlipidemia, and cardiovascular disorders [11]. Former studies have showed that BER could lessen experimental colitis through improving epithelial barrier function, inhibiting lipid peroxidation, elevating the levels of tight junction proteins, and balancing the intestinal microbiota [12]. Remarkably, BER has been demonstrated to ameliorate the obesity and insulin resistance through modulation of intestinal microbiota [13].

Although the anticolitis effect of BER was studied by Yan et al. [14], the underlying mechanism of BER in UC is still a continuing topic that gains the attention of many researchers. Furthermore, in the study of Yan et al. [14], the antioxidant efficacy of BER was not examined in addition to the ability of BER to enhance the Nrf2/HO-1 pathway as well as suppress the MAPK/PGE2 signaling pathway. Hence, to better understand the anticolitis efficacy of BER, the current study spotlights its potential to attenuate the experimental colitis involving Nrf2/HO-1 and MAPK/PGE2 signaling pathways. Further, the effect of berberine on apoptosis and oxidative stress and its underlying mechanisms of action were also investigated.

## 2. Materials and Methods

### 2.1. Chemicals

Berberine chloride hydrate (CAS Number: 633-65-8) was bought from Sigma (St. Louis, MO, USA).

### 2.2. Experimental Animals and Ethical Statement

The study was conducted on thirty-five adult male Wistar rats (200–225 g body weight). The experimental procedures were performed according to the National Institutes of Health (NIH) Guidelines for the Care and Use of Laboratory Animals, 8^th^ edition (NIH Publication No. 85-23, revised 1985). Also, the guidelines of the Department of Zoology, Faculty of Science, Helwan University (Cairo, Egypt) approved our experimental protocol and animal handling (Authorize Number: HU2019/Z/AE0219-06). Experimental rats were kept under monitored environmental conditions (24 ± 2°C temperature and 12 h/12 h light/dark cycle). Experimental animals were raised on standard rodent food and provided water *ad libitum*. Before induction of colitis, animals were deprived from food overnight, but water was received freely.

### 2.3. Induction of UC in Rats

Following the fasting overnight, rats were exposed to light ether anesthesia. Colitis was induced in rats as stated by Almeer et al. [1]. Briefly, colons lavage was done by 2 mL of saline and the lower abdomen was palpated to discard the remaining fecal matter. After that, intrarectal injection of 2 mL acetic acid (AcOH, 4% *v*/*v* in 0.9% saline) was performed by inserting a polyurethane cannula (2 mm in diameter) into the anal opening for 6 cm for 30 seconds. Animals were kept in an inverted position and head down for two minutes to avoid loss of AcOH inside the colon.

### 2.4. Experimental Protocol and BER Treatment

Randomly, rats were allocated into five groups (7 rats per each group) as follows:

- Group I (CNTR): rats treated with saline orally (2 mL, 0.9% NaCl solution)
- Group II (BER): rats received 300 *μ*L berberine by oral gavage once daily for seven days at a dose of 50 mg/kg following the method of Zhu et al. [15]
- Group III (UC): rats with UC received saline orally
- Groups IV (BER-25+UC): rats with UC and administered with low dose of BER (25 mg/kg) orally
- Group V (BER-50+UC): rats with UC received BER at high dose (50 mg/kg) orally

All previously mentioned treatments were received every day for 7 successive days before UC induction by means of intrarectal AcOH injection. After 12 h from AcOH administration, animals were killed with minimal pain sensation. Colon samples (7-8 cm in length) were weighed, gently washed by saline, and imaged by a Samsung camera (WB30F, Japan). Portions from colon samples (1 cm) were directly fixed with 10% neutral buffered formalin and embedded in paraffin blocks for both histopathological and immunohistochemical examinations. The other colon portions were kept at -70°C for conducting biochemical and molecular tests.

### 2.5. Morphology and Histopathological Evaluation

Each colon specimen was washed by cold normal saline (0.9%), and the macroscopic visible injure was scored according to Almeer et al. [1] as follows: no ulcerations (0 points), hyperemia with no ulcers (1 point), slight mucosal edema and bowel wall thickening (2 points), moderate edema with erosions at one site (3 points), severe ulceration at more sites less than 5 mm (4 points), and extreme ulcerations at more sites greater than 5 mm (5 points). After that, colon tissue samples were embedded in paraffin blocks and sectioned (4–5 *μ*m thickness) followed by hematoxylin and eosin (H&E) staining for histopathological screening. The histological injure was scored and given grades as follows: 0–3 for the prominent inflammation and infiltration of inflammatory cells, 0–3 for the inflammation extended to mucosa, submucosa, and transmural layers, and 0–4 for crypt damage with coagulative necrosis. The sum of all evaluated parameters represented the total histological score [16].

### 2.6. Immunohistochemical Studies

Colon sections (4-*μ*m-thickness) were incubated in 10% normal serum containing 1% bovine serum albumin in Tris-buffered saline for 2 hours at 25°C followed by incubation of tissues with 1% H_2_O_2_ for 15 minutes. They were incubated overnight with primary monoclonal antibody against TGF-*β* (Cat #: MA5-16949; Invitrogen, Thermo Fisher Scientific corporation, Waltham, Massachusetts, USA) at 4°C. Rabbit anti-mouse secondary antibody that is labeled by biotin (Dako system kit) was added followed by avidin/biotin-peroxidase detection solution (Dako system kit). The colon samples were stained with hematoxylin and then dehydrated and mounted using Aquatex fluid (Merck KGaA, Darmstadt, Germany).

### 2.7. Oxidative Stress Markers

Tissue homogenates from colon samples were prepared by mixing the tissues with 50 mmol of Tris–HCl buffer (pH 7.4). The protein contents in the supernatants were calculated based on the Lowry method [17]. Lipid peroxidation was assessed in the obtained supernatants via measurement of malondialdehyde (MDA) following the method described by Ohkawa et al. [18]. Nitrite/nitrate (nitric oxide, NO) contents were determined following the protocol of Green et al. [19]. Further, the level of reduced glutathione (GSH) was assessed based on the method stated by Sedlak and Lindsay [20].

### 2.8. Antioxidant Enzymatic Activities

Enzymatic activity of superoxide dismutase (SOD) in the supernatants was measured building on the method of Nishikimi et al. [21]. The activity of catalase (CAT) was assessed following the protocol of Aebi [22]. Additionally, glutathione peroxidase (GPx) activity was measured spectrophotometrically as formerly mentioned by Paglia and Valentine [23]. The activity of GPx was detected via the reduction in NADH per minute depending on the reaction with glutathione reductase (GR). Further, glutathione reductase (GR) activity was calculated by glutathione-dependent oxidation of NADPH at 340 nm and presented as U/mg protein [24].

### 2.9. Colonic Myeloperoxidase Activity and Cytokine Concentrations

Myeloperoxidase (MPO) activity was measured as an indicator for neutrophil infiltration into the inflamed colonic mucosa as stated by Bradley et al. [25] with minor modifications. Colon homogenates were undergone to three freeze-thaw cycles and then centrifuged at 10,000 ×g for 10 min at 4°C. The supernatant (0.1 mL) was mixed with 2.9 mL of 0.05 M phosphate buffer (pH 6) and 1 mL of 1.6 mM o-dianisidine hydrochloride containing 0.0005% (*v*/*v*) H_2_O_2_. The MPO activity was assessed by the change in the absorbance at 460 nm and presented as U/mg protein. Prostaglandin E2 (PGE2; Cat #: KGE004B), interleukin-1 beta (IL-1*β*; Cat #: RLB00), IL-6 (Cat #: R6000B), and tumor necrosis factor alpha (TNF-*α*; Cat #: RTA00) levels were detected by using the ELISA kit (R&D System, Minneapolis, MN, USA), based on the manufacturer's information and expressed as pg/mg protein.

### 2.10. Quantitative Real-Time PCR (qRT-PCR) Analysis

Extraction of total RNA and synthesis of first-strand cDNA were performed following the protocol of Abdel Moneim [26]. The expressions of nuclear factor (erythroid-derived 2)-like 2 (Nrf2; *Nfe2l2*), heme oxygenase-1 (*Hmox1*), inducible nitric oxide synthase (iNOS; *Nos2*), and p38 mitogen-activated protein kinases (p38 MAPK; *Mapk14*) were assessed by qRT-PCR technique using an Applied Biosystems 7500 Instrument. The program was as follows: 95°C for 4 min, followed by 40 cycles of 94°C for 60 s and 55°C for 60 s, and finally, extension step at 72°C for 10 min. After PCR amplification, the ΔCt from triplicate experiments was calculated in respect to the content of the housekeeping gene, glyceraldehyde 3-phosphate dehydrogenase (*Gapdh*, Ct). The sequences of primers used for detecting different genes are listed in Table 1.

### 2.11. Statistical Analysis

All represented data are displayed as the means ± standard deviation (SD). Using the statistical package SPSS, version 17.0, one-way analysis of variance (ANOVA) was utilized to analyze the data followed by Duncan's multiple comparison test to differentiate between groups. Differences between nonnormally distributed data (macroscopic and histological scores) were analyzed using the Kruskal–Wallis test, followed by the Dunn multiple comparison test. Statistical difference was achieved when *p* values are less than 0.05.

## 3. Results

### 3.1. BER Mitigated the Colonic Macroscopic and Histopathological Changes in Rat Colitis

**Figure 1:**
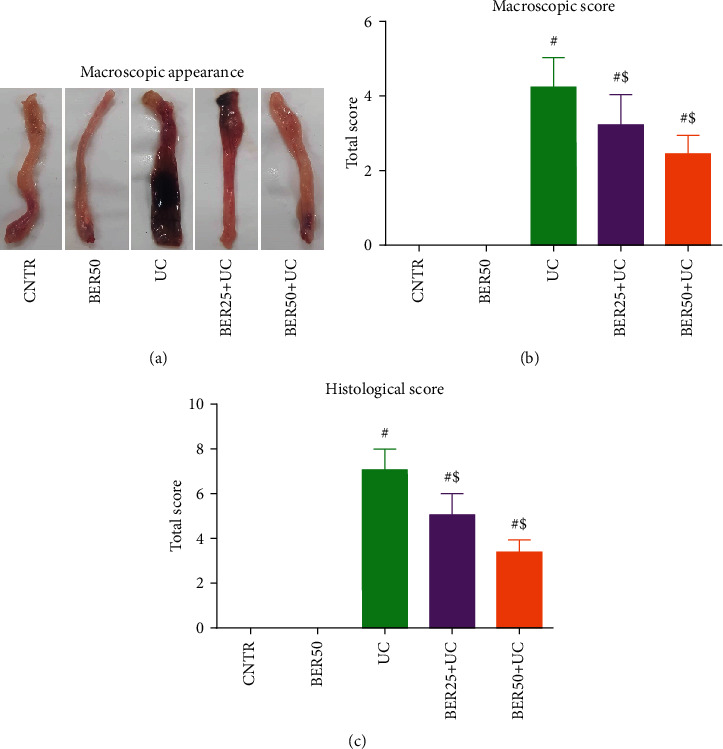
Effect of berberine at different doses on experimental colitis in rats. (a) Macroscopic appearance of rat colon in experimental colitis. (b) Macroscopic score. (c) Histological score. The results are expressed as the mean ± SD (*n* = 7). ^#^*p* < 0.05 compared to the CNTR group (intact group); ^$^*p* < 0.05 compared to UC-untreated group.

Macroscopic picture of experimental colitis revealed transmural inflammation of the colon accompanied by signs of hyperemia, necrosis, corrosion, mucosal edema, and ulcerations (Figure 1(a)). However, a seven-day administration of BER at low (25 mg/kg) and high (50 mg/kg) doses (*p* < 0.05) showed less severe necrosis, corrosion, and ulceration with a significantly decreased in the degree of colonic injure, by about 28% and 43% correspondingly in comparison with the UC-untreated rats (Figure 1(b)). Consistent with the microscopic scores, a significant decline was found in the histopathologic score in groups administered with 25 and 50 mg/kg of BER; the rats in these groups exhibited less mucosal damage compared with UC-untreated rats (Figure 1(c)).

**Figure 2:**
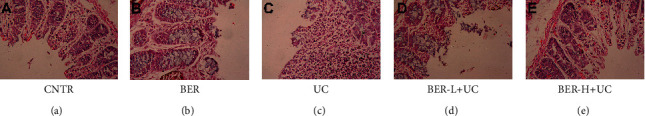
Histological findings of the colon (H&E). (a) Colon from the CNTR group (intact group). (b) Colon from the BER group (without ulceration rats). (c) Colon in experimental colitis, untreated group; tissue injury is characterized by severe mucosal damage with severe hemorrhage and infiltration of inflammatory cells. (d) Colon in BER low dose (25 mg/kg) and experimental colitis, treated group, showing reduced tissue injury. (e) Colon in BER high dose (50 mg/kg) and experimental colitis, treated group, showing the greatest reduction in the tissue injury characterized by less mucosal damage and low infiltration of inflammatory cells. All the images were taken at a 200x magnification.

Furthermore, control and BER-treated alone groups showed normal mucosal epithelium of tall columnar epithelial cells with goblet cells (Figures 2(a) and 2(b)). However, the histological evaluation of the UC-untreated rats showed severe necrosis, mucosal edema, focal infiltration with inflammatory cells, erosion of the mucosal layer with subsequent desquamation and loss of epithelial layer, and crypt damage (Figure 2(c)). BER reduced the degree of inflammation, infiltration, and crypt damage in a dose-dependent manner (Figures 2(d) and 2(e)). Interestingly, BER at high dose (50 mg/kg) showed intact organization of the colonic mucosa, tidy arrangement of the epithelium, and typical shape of the crypts.

### 3.2. BER Restored the Colon Oxidative Damage in Rat Colitis

**Figure 3:**
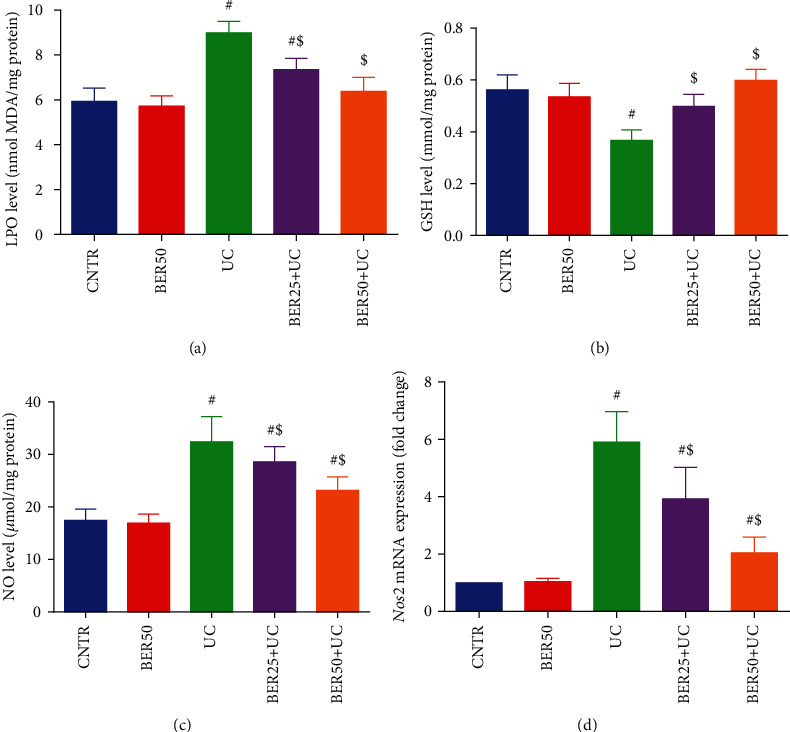
Effect of berberine at different doses on oxidative stress markers ((a) LPO, (b) GSH, and (c) NO) and (d) *Nos2* mRNA expression in colonic tissue of experimental colitis in rats. The results are expressed as the mean ± SD (*n* = 7). qRT-PCR results of *Nos2* were normalized with *Gapdh* and represented as fold change as compared to mRNA levels in the CNTR rats. ^#^*p* < 0.05 compared to the CNTR group (intact group); ^$^*p* < 0.05 compared to UC-untreated group.

Regarding the nonenzymatic oxidant molecules in colon homogenate, AcOH intrarectal injection caused marked elevations (*p* < 0.05) in MDA (Figure 3(a)) and NO levels (Figure 3(b)) in colon homogenate when compared to control animals. Additionally, there is notable upregulation in the mRNA expression of *Nos2* (Figure 3(d)) in the experimental colitis group in respect to control group. Adversely, GSH content (Figure 3(c)) in colon homogenate showed marked decline (*p* < 0.05) after injection of rats with AcOH in comparison with control rats. However, daily treatment with BER at low (25 mg/kg) and high (50 mg/kg) doses for seven days exhibited noteworthy improvement in the colon mucosal oxidative injury, evidenced by the marked increase in GSH level (*p* < 0.05) together with notable decreases (*p* < 0.05) in MDA and NO levels in respect to experimental colitis group. Also, noticeable downregulation (*p* < 0.05) was detected in *Nos2* expression in BER-treated rats compared to UC group (Figure 3). The obtained results mentioned that BER treatment alone has no effect on the tested markers.

**Figure 4:**
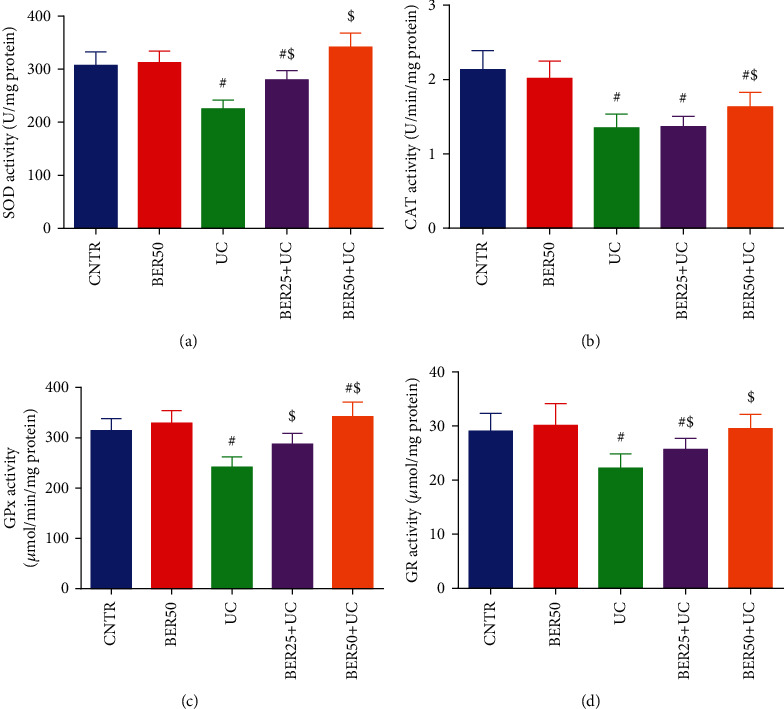
Effect of berberine at different doses on antioxidant enzymes activity ((a) SOD, (b) CAT, (c) GPx, and (d) GR) in colonic tissue of experimental colitis in rats. The results are expressed as the mean ± SD (*n* = 7). ^#^*p* < 0.05 compared to the CNTR group (intact group); ^$^*p* < 0.05 compared to UC-untreated group.

For the colon antioxidant enzymatic activities, Figure 4 presented notable suppressions (*p* < 0.05) in SOD (Figure 4(a)), CAT (Figure 4(b)), GPx (Figure 4(c)), and GR (Figure 4(d)) activities in experimental colitis group when compared to the control group. Treatment with BER (low (25 mg/kg) and high (50 mg/kg) doses) significantly (*p* < 0.05) enhanced the antioxidant activity of tested enzymes in colon mucosa as compared to experimental colitis group indicating the antioxidant potential of BER against the experimental colitis-induced damage (Figure 4).

**Figure 5:**
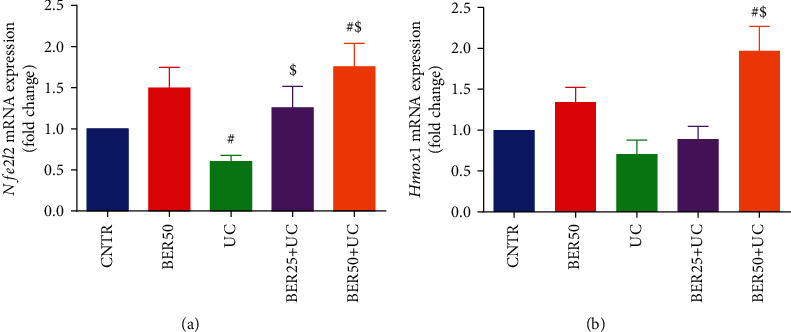
Effect of berberine at different doses on (a) *Nfe2l2* and (b) *Hmox1* mRNA expression in colonic tissue of experimental colitis in rats. The results are expressed as the mean ± SD (*n* = 7). qRT-PCR results were normalized with *Gapdh* and represented as fold change as compared to mRNA levels in the CNTR rats. ^#^*p* < 0.05 compared to the CNTR group (intact group); ^$^*p* < 0.05 compared to UC-untreated group.

To illustrate the molecular mechanism of antioxidant capacity of BER in rats with experimental colitis, quantitative RT-PCR analysis of the Nrf2/HO-1 signaling was assessed in colon tissues (Figure 5). The mRNA expression of *Nfe2l2* (Figure 5(a)) displayed significant downregulation (*p* < 0.05) in experimental colitis group in comparison with control group. Rats received BER at low (25 mg/kg) and high (50 mg/kg) doses displayed marked upregulations (*p* < 0.05) in *Nfe2l2* expression in respect to experimental colitis group. However, relative to control untreated group, *Hmox-1* expression (Figure 5(b)) in colon mucosa of experimental colitis group did not show any significant difference, and BER administration at high dose only elevated (*p* < 0.05) *Hmox1* expression in the rat colon in respect to the experimental colitis group.

### 3.3. BER Lessened Colonic Inflammatory Markers in Rat with Colitis

**Figure 6:**
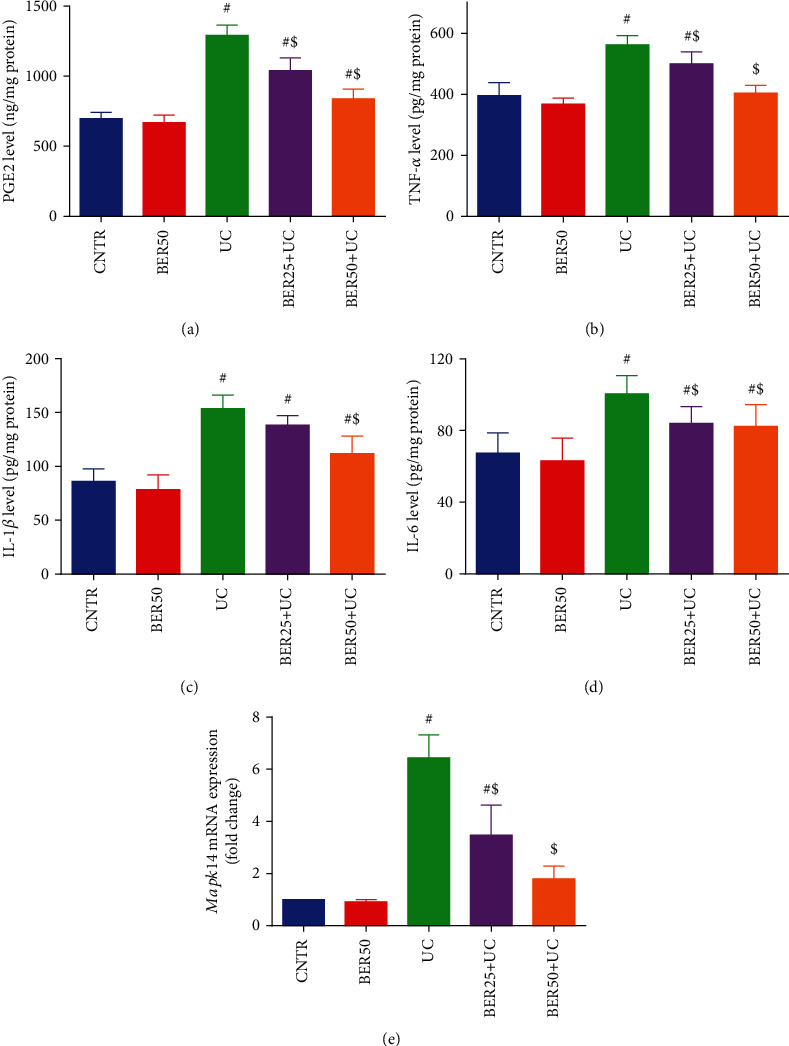
Effect of berberine at different doses on inflammatory mediators and cytokines ((a) PGE2, (b) TNF-*α*, (c) IL-1*β*, and (d) IL-6) and (e) *Mapk14* mRNA expression in colonic tissue of experimental colitis in rats. The results are expressed as the mean ± SD (*n* = 7). qRT-PCR results of *Mapk14* were normalized with *Gapdh* and represented as fold change as compared to mRNA levels in the CNTR rats. ^#^*p* < 0.05 compared to the CNTR group (intact group); ^$^*p* < 0.05 compared to UC-untreated group.

Because of the chronic inflammatory reaction and excess cytokine secretion that are associated with UC, IL-1*β*, IL-6, TNF-*α*, and PGE2 levels were assessed in colon mucosa as well as the mRNA expression of *Mapk14*. As illustrated in Figure 6, prominent rises in levels of TNF-*α* (Figure 6(a)), IL-1*β* (Figure 6(b)), IL-6 (Figure 6(c)), and PGE2 (Figure 6(d)) were detected in experimental colitis group along with significant upregulation in the mRNA expression of *Mapk14* (Figure 6(e)) in the experimental colitis group compared to the control group. Adversely, treatment with BER noticeably reversed (*p* < 0.05) these elevations induced by AcOH indicating that BER efficiently mitigated the colonic inflammation and mucosal injury in experimental colitis rat model.

**Figure 7:**
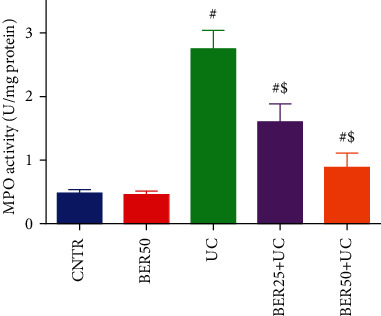
Effect of berberine at different doses on MPO activity in colonic tissue of experimental colitis in rats. The results are expressed as the mean ± SD (*n* = 7). ^#^*p* < 0.05 compared to the CNTR group (intact group); ^$^*p* < 0.05 compared to UC-untreated group.

In the present study, the activity of MPO was significantly elevated in the colonic tissues of experimental colitis (*p* < 0.05) compared to the control rats. Nevertheless, BER treatment meaningfully suppressed MPO activity gradually in a dose-dependent manner in the colon (Figure 7).

**Figure 8:**
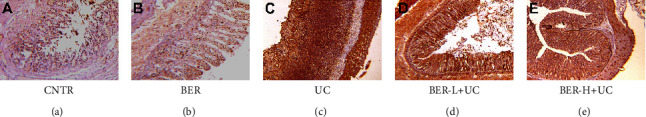
Berberine supressing the protein expressions of TGF-*β* in colonic tissue of experimental colitis in rats. (a) Colon from the CNTR group (intact group). (b) Colon from the BER group (without ulceration rats). (c) Colon in experimental colitis, untreated group. (d) Colon in BER low dose (25 mg/kg) and experimental colitis, treated group. (e) Colon in BER high dose (50 mg/kg) and experimental colitis, treated group. All the images were taken at a 100x magnification.

Further, the expression of TGF-*β* was detected by immunohistochemistry. As shown in Figure 8(c), the immunohistochemistry staining of TGF-*β* in UC-untreated colon showed stronger immunoreactivity than that in the control (Figure 8(a)) and BER (Figure 8(b)) groups. Meanwhile, the BER treated UC model at low (Figure 8(d)) and high (Figure 8(e)) doses; the immunohistochemistry staining of TGF-*β* in UC-untreated colon showed low immunoreactivity than that in the UC-untreated group.

### 3.4. BER Attenuated Colonic Apoptotic Biomarkers in Rat Model with Colitis

**Figure 9:**
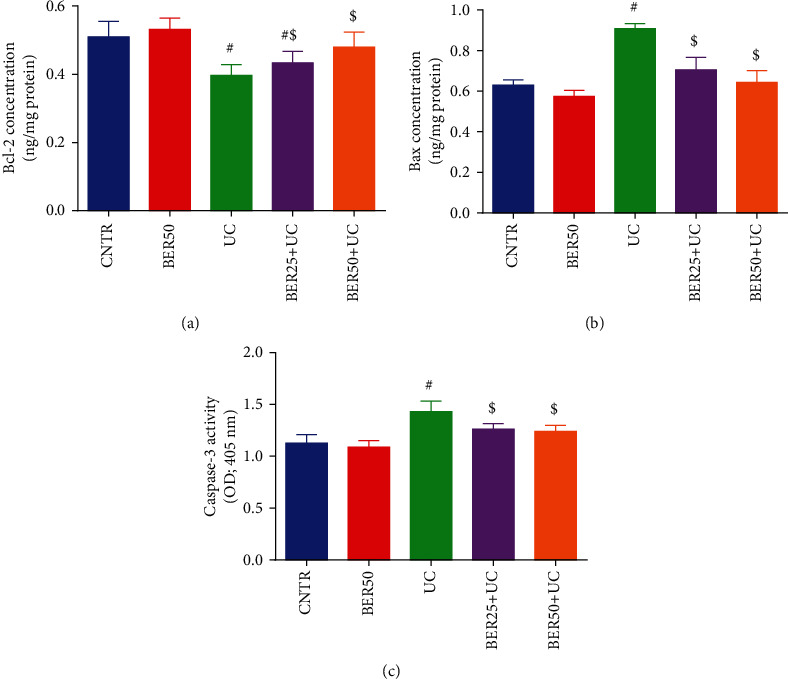
Effect of berberine at different doses on apoptosis-related protein ((a) Bcl-2, (b) Bax, and (c) caspase-3) in colonic tissue of experimental colitis in rats. The results are expressed as the mean ± SD (*n* = 7). ^#^*p* < 0.05 compared to the CNTR group (intact group); ^$^*p* < 0.05 compared to UC-untreated group.

The contents of Bcl-2 (Figure 9(a)), Bax (Figure 9(b)), and caspase-3 (Figure 9(c)) were estimated in the colon tissues of treated and untreated UC model groups. In comparison with the control group, notable increases (*p* < 0.05) in colonic proapoptosis proteins (caspase-3 and Bax) accompanied by marked decline (*p* < 0.05) in antiapoptotic protein (Bcl-2) were detected in rats with experimental colitis. When BER was administered, noteworthy declines (*p* < 0.05) were observed in caspase-3 and Bax levels with augmentation (*p* < 0.05) in Bcl-2 level compared to experimental colitis group. These findings suggested that BER had significant impact on colonic cell apoptosis in rats with experimental colitis.

## 4. Discussion

UC as a subtype of inflammatory bowel disease is characterized by recurring inflammation and ulceration of the colon. It is assumed that the pathogenesis of UC is based on several factors for induction of colitis such as genetic reasons, age, sex, and environmental factors [27]. The intestinal epithelial lining acts as a physical barrier that is designed by a tight continuous layer of epithelial cells. Disturbance of such tight junctions gives rise to further inflammatory reactions and oversecretions of chemokines and adhesive molecules [28]. Additionally, elevated levels of inflammatory cytokines exaggerate the innate and acquired immune reactions, involving activation of inflammatory CD4^+^ T cells [29].

Immune modulating, anti-inflammatory medications and steroids have been widely used for alleviation of UC. Unfortunately, these medications have undesirable effects such as anemia, vomiting, and general edema. Therefore, researchers are trying to find natural compounds existing in traditional herbs. Previously, these natural compounds showed potent anti-inflammatory effects and effectively treat chronic inflammatory conditions [15].

The existing investigation outlined the anticolitis potency of BER to alleviate experimental colitis in rat experimental model. Such palliative effect of BER was evidenced by histological screening of the colon. Intrarectal injection of rats with AcOH resulted in prominent disruption of the colonic mucosal cells indicated macroscopically. The colon photomicrograph displayed severe necrosis, mucosal edema, focal inflammatory cell infiltration, and erosion of the mucosal layer in the experimental colitis group. These results were in accordance with previous studies [5, 30]. However, the animals treated with BER had less colonic mucosal injury that was evidenced by noticeable diminishing in the aforementioned alterations, and this is in line with an earlier report [31].

Inflammatory response is a characteristic feature of intestinal barrier disruption and is a hallmark in the pathogenesis of UC [32]. Our results demonstrated that AcOH intracolonic injection resulted in significant elevations in IL-1*β*, IL-6, TNF-*α*, and PGE2 levels. Our findings are inconsistent with Yan et al. [14] who found that BER effectively restrained dextran sulfate sodium- (DSS-) induced intestinal damage and colitis in mice. Gut inflammation stimulates colonic macrophages with subsequent oversecretion of inflammatory cytokines, such as TNF-*α* and interleukins (IL-1*β*, IL-6, and IL-4) which further progress UC through production of destructive enzymes and free radicals [9]. Inflammatory cytokines are involved in modifying mucosal immune system as the neutrophils and macrophages that disrupt the epithelial integrity with consequent colon injury [7]. Furthermore, marked elevation was detected in MPO activity in experimental colitis group. MPO is an enzyme produced by neutrophils and stimulates the generation of reactive oxygen species. Accordingly, the MPO activity is proportional to the infiltration of neutrophils in colonic mucosa and can be used as an index to evaluate the severity of UC [33].

TGF-𝛽 is a cytokine with potent inflammatory activity that can promote angiogenesis and suppress immune responses and was reported to promote growth, invasion, and metastasis of cancer [34]. It is expressed in gut epithelial cells, fibroblasts cells, and T-cells. Significant high level of TGF-𝛽1 was reported in an ulcerative colitis rat model, and its level was correlated with UC disease severity [35].

Prostaglandins are synthesized from arachidonic acid by cyclooxygenase enzymes [36]. They may elevate vascular permeability and encourage leukocyte chemotaxis, resulting in inflammatory cell infiltration, colonic mucosal inflammation, tissue damage, edema, and ulceration [28]. Notable increment was observed in PGE2 in experimental colitis group. Steroids that inhibit the release of arachidonic acid as well as nonsteroidal anti-inflammatory drugs that block or inhibit COX function are effective therapy for treatment of inflammation [6]. MAPKs (extracellular regulated kinase, c-Jun amino-terminal kinase, and P38) are the signalling molecules that have a pivotal role in the course of UC. They are involved in regulation of proinflammatory cytokines such as TNF-*α*, IL-1*β*, and IL-6 as well as the anti-inflammatory cytokine as IL-10. p38 MAPK are implicated in regulation of inflammatory factors, growth factors, and cell stress factors [37]. Our findings showed significant upregulation in mRNA expression of *Mapk14* in experimental colitis group. Previous reports revealed high activity of MAPKs during IBD intestinal epithelial injury [1, 9].

Accumulating evidences suggested that BER alleviated the symptoms of chronic inflammation in many diseases such as allergic conditions, hepatitis, gastroenteritis, and experimental autoimmune encephalomyelitis [15]. Zhang et al. [11] stated that BER restored the intestinal barrier function in experimental colitis induced by dextran sodium sulfate in mice through upregulation of expression of tight junction proteins (zonula occluden-1, occludin, and epithelial cadherin) in colonic tissue. Further, BER exhibited noteworthy suppression of the UC-associated inflammatory response on lamina propria CD4^+^ T cells via activation of AMP-activated protein kinase and inhibition of oxidative phosphorylation [29]. In the present study, treatment of rats with BER significantly alleviated the colonic inflammation due to suppression of overproduction of MPO and inflammatory cytokines such as TNF-*α*, IL-1*β*, and IL-6. Additionally, Lee et al. [38] found marked downregulation of mRNA expression of COX-2 in colonic tissue of mice with UC induced by 2,4,6-trinitrobenzene sulfonic acid. Therefore, the reduction of PGE2 level in colonic mucosa after BER administration to colitis group may refer to its inhibitory effect on COX enzymatic action. Also, BER also maintained the balance between proinflammatory mediators as interleukins and anti-inflammatory factors evidenced by increasing the protein expression of TGF-*β* in colon samples. Significant downregulation of p38 MAPK signalling pathway was also noticed in BER-treated group, and this contributes to its anti-inflammatory responses which paralleled the histopathological evidence of its protection. MAPK signalling pathway affects the balance of inflammatory cytokines and influences the inflammatory process, thus preserving the health of the gastrointestinal tract.

Oxidative stress and exhaustion of cellular antioxidant defence are well known to be involved in the pathophysiology of UC [39]. In accordance with the former authors, oxidative damage and overproduction of free radicals were reported in colitis-induced experimental model [1]. Such hazardous free radicals exacerbate the intestinal tissue damage and ulcer formation [28]. Our results revealed marked decline in GSH and suppressions of antioxidant enzymes paralleled with increase of MDA and NO levels in UC group. Zheng et al. [40] stated the enhancement of oxidative stress by the excess secretion of MPO and proinflammatory cytokines such as TNF-*α* and IL-6 in colitis. SOD overwhelms excess superoxide radicals via its conversion to hydrogen peroxide (less toxic metabolite) and water, but CAT acts by conversion of hydrogen peroxide to water and molecular oxygen, so they provide mutual significant protection against reactive oxygen species. GSH is a vital cofactor to overcome the harmful peroxides generated from oxygen radical. Under inflammatory situations, GSH level ensuing in severe injury to colon mucosa was diminished [41]. MDA is an indicator for peroxidation of lipids after attack of lipid molecules by unstable free radicals [7]. In addition, the level of NO as well as the expression of iNOS in colonic mucosa displayed significant elevations in the active period of UC [42]. The current results revealed that BER had the ability to attenuate the colonic oxidative damage induced in experimental colitis rats, possibly by reaction with the generated free radicals and lessening the peroxidation of cellular lipid by scavenging lipid peroxyl radicals. Significant rises were detected in the enzymatic activities of SOD and CAT in colon and serum samples in dextran sodium sulfate-induced experimental colitis in mice compared to the control [11].

Among the possible mechanisms involved in the protective efficiency of BER in UC is regulating the Nrf2/HO-1 signalling pathways. Nrf2 is a cytoprotective transcription factor that activates the antioxidant enzymatic activities to protect the cells against damage induced by oxidative stress. In the cytoplasm, it is attached to the Kelch-like erythroid cell-derived protein 1 (Keap1). When the cell becomes under oxidative stress conditions, it moves to the nucleus, binds with the antioxidant response element, and stimulates HO-1 [43]. Previous reports stated that BER activated markedly Nrf2 nuclear translocation in different disease models [44–46]. Thus, in the current study, the antioxidant effect of BER in the colon was also illustrated by its potent activation to Nrf2/HO-1 pathway in experimental colitis in rats.

Many inflammatory cytokines in the intestine can trigger the apoptotic changes in epithelial cells of the intestine which in turn damage the tight junctions and alter the mucosal barrier homeostasis [42]. Earlier reports illustrated that apoptosis of intestinal epithelial cells is implicated in the progression of UC. Additionally, lessening gastrointestinal apoptosis was helpful to great extent in the recovery of UC through restoring gut homeostasis and immunity [47]. Turner et al. [48] found that BER and its biological derivative, dihydroberberine, could activate AMP-activated protein kinase (AMPK) through suppression of mitochondrial respiratory complex I. Further, marked inhibition of colorectal tumorigenesis was reported in BER-administered mice by Li et al. [49] as a result for activation of AMPK. It is well known that AMPK acts as a key regulator for cellular energy metabolism in addition to vital cellular processes, as cell proliferation, differentiation, and survival [50]. Therefore, the AMPK activation contributes to the soothing effect of BER to mitigate UC-associated intestinal epithelial damage. Herein, we observed that BER reversed the apoptotic changes induced in experimental colitis by lessening the production of caspase-3 and Bax as well as elevating Bcl-2 level in colon mucosa. Hence, it indicated that BER could improve the worse intestinal apoptosis situation, and diminishing the apoptosis in intestinal epithelial cells is considered as an additional anticolitis effect of BER in an experimental colitis rat model.

## 5. Conclusion

In summary, the present study demonstrated that BER improved colitis-induced injury in colon mucosa through its antioxidant and anti-inflammatory actions possibly via the Nrf-2 and p38 MAPK signaling pathways. The promising regulatory role of BER on these signaling pathways might support its use as an effective candidate for treatment of UC. However, the underlying mechanisms regarding the impact of BER on UC-associated inflammatory, fibrotic, tumorigenic events, and proliferation and differentiation of intestinal stem cells warrant further investigations.

## Figures and Tables

**Table 1:** Primer sequences of genes analyzed in real-time PCR.

Name	Forward primer (5′---3′)	Reverse primer (5′---3′)
*Gapdh*	AGTGCCAGCCTCGTCTCATA	GATGGTGATGGGTTTCCCGT
*Nfe2l2*	TTGTAGATGACCATGAGTCGC	ACTTCCAGGGGCACTGTCTA
*Hmox1*	GCGAAACAAGCAGAACCCA	GCTCAGGATGAGTACCTCCCA
*Nos2*	GTTCCTCAGGCTTGGGTCTT	TGGGGGAACACAGTAATGGC
*Mapk14*	AGAGTCTCTGTCGACCTGCT	GGGTCGTGGTACTGAGCAAA

The abbreviations of the genes: G*apdh*: glyceraldehyde-3-phosphate dehydrogenase; *Nfe2l2*: nuclear factor erythroid 2-related factor 2; *Hmox1*: heme oxygenase 1; *Nos2*: inducible nitric oxide synthase; *Mapk14*: p38 mitogen-activated protein kinases.

## Data Availability

All relevant data are within the paper.
